# Zearalenone-Induced Mechanical Damage of Intestinal Barrier via the RhoA/ROCK Signaling Pathway in IPEC-J2 Cells

**DOI:** 10.3390/ijms232012550

**Published:** 2022-10-19

**Authors:** Biying Huang, Jingjing Wang, Aixin Gu, Tianhu Wang, Jianping Li, Anshan Shan

**Affiliations:** Institute of Animal Nutrition, Northeast Agricultural University, Harbin 150030, China

**Keywords:** zearalenone, RhoA/ROCK pathway, intestinal barrier, IPEC-J2 cells

## Abstract

Zearalenone (ZEN) is a widespread contaminant of cereals and agricultural products which causes food safety issues. Ingesting food or feed contaminated with ZEN can disrupt the intestinal epithelial barrier function. The RhoA/ROCK signaling pathway plays a key role in regulating the epithelial barrier function, but studies on such roles have rarely focused on the intestine. The aim of this experiment was to investigate the exact mechanism of ZEN-induced intestinal barrier damage and whether the RhoA/ROCK signaling pathway is involved. The results showed that ZEN significantly induced alkaline phosphatase (AP) activity and FITC–dextran (4 kDa) passage across the epithelial barrier, which significantly reduced the transepithelial resistance (TEER). Meanwhile, ZEN could induce the significantly down-regulated mRNA expression of tight junction proteins (occludin, claudin-1, ZO-1, and claudin-3) and redistribution of ZO-1 immunofluorescence. Further studies demonstrated that ZEN exposure activated the RhoA/ROCK signaling pathway, significantly up-regulated the mRNA expression of ROCK1, the main effector of the signaling pathway, the protein expression of phosphorylated myosin light chain (MLC) and myosin light chain kinase (MLCK), and relatively increased the activity of ATP in cells, simultaneously remodeling the cytoskeleton (F-actin). Overall, our study indicated that ZEN induced intestinal barrier dysfunction by activating the RhoA/ROCK signaling pathway.

## 1. Introduction

Zearalenone (ZEN) is a nonsteroidal estrogenic fungal toxin produced by *Fusarium oxysporum* [1]. It is widely present in the environment as a contaminant that has been found in agricultural products and crops worldwide. [2,3,4]. Consequently, the toxic effects and mechanisms of ZEN have been extensively studied by researchers all over the world. A number of studies have suggested that ZEN can cause damaging effects on a wide range of tissues and cells [5,6,7]. Of concern is the extreme vulnerability of mammalian enterocytes to the effects of mycotoxins in the gut [8]. In previous studies, we have demonstrated that reactive oxygen species (ROS) production and accumulation in PK15 cells and SIEC02 cells are induced by ZEN, causing changes in the activities of oxidative stress-related enzymes such as GR and MDA, and ultimately apoptosis [9,10]. Most studies have focused on the estrogen toxicity, genotoxicity, immunotoxicity, and carcinogenesis of the damage caused by ZEN exposure [11,12]. However, studies on the mechanisms of ZEN-induced intestinal damage are relatively scarce.

The gastrointestinal tract is the primary defense against the stimulation of food contaminants [13]. When humans and animals ingest food containing mycotoxins, the epithelial cell layer and the intestine are exposed to toxic substances, which definitely impose negative effects on intestinal health and further cause an immune response in the gut [14]. Significantly, tight junctions (TJs) play an instrumental part in the mechanical immune barrier in the intestine [15]. It consists of a complex structure of multiple transmembrane proteins that have a function in the maintenance of epithelial barrier function and the regulation of gut mucosal permeability [16].

RhoA has been shown to play a key role in the regulation of tight junctions in the gut [17,18]. RhoA is a critical regulatory factor that regulates the integrity of tight junctions between cells and is also involved in the regulation of many other vital cellular processes including actin cytoskeleton organization, proliferation and apoptosis, remodeling of the extracellular matrix, and smooth muscle cell contraction [19]. It is a serine/threonine kinase in the AGC family [20]. The activation of RhoA and its downstream targets leads to alterations in tight junction proteins, altering the permeability of the intestinal epithelium [21]. Many studies have demonstrated that the downstream targets of RhoA regulation are mainly ROCKs and that ROCKs have two homologues: ROCK1 and ROCK2 [22]. They can directly phosphorylate myosin light chains (MLC) and release energy simultaneously [23]. The energy produced causes the cytoskeletal actin filament (F−actin) to slide, and cells contract to form the intercellular space, resulting in an increase of intercellular permeability [24,25]. Although the RhoA/ROCK signaling pathway regulates the epithelial barrier function, most of the studies have mainly focused on the optic nerve, brain, cardiovascular system, and cancer. There are few studies on this pathway to regulate the intestinal barrier.

The purpose of this paper is to elucidate the mechanical damage caused by ZEN to the intestine and its related mechanism. More importantly, our investigations will identify the mechanism of the action of the RhoA/ROCK signaling circuit in the pathological disruption of ZEN-mediated tight junctions and intestinal barrier dysfunction. Overall, the findings of this work provide valuable input for the prevention and treatment of human intestinal diseases associated with ZEN.

## 2. Results

### 2.1. Measurement of Transepithelial Electrical Resistance (TEER)

**Figure 1:**
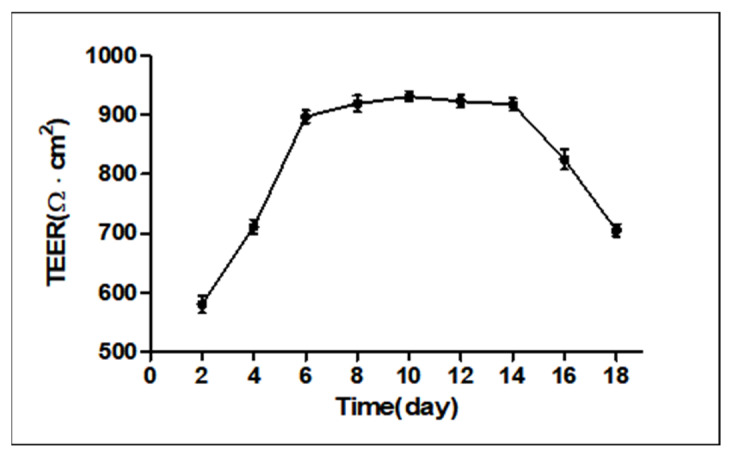
IPEC-J2 cell monolayers. Cells were grown and differentiated on Transwell^®^ polyester membranes.

As shown in Figure 1, we measured TEER values from days 1 to 18 and the TEER remained stable from day 6 to day 14, indicating that the monolayer confluence model was successfully established.

### 2.2. Effects of ZEN on Permeability Barrier Function and Intestinal Integrity

#### 2.2.1. Effect of ZEN on Intestinal Epithelial Cell Viability

**Figure 2:**
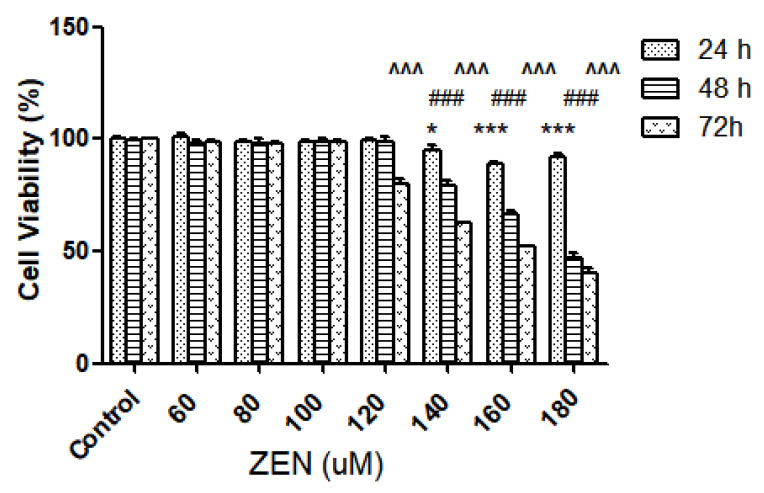
Effects of different concentrations and times of incubation of ZEN on the toxicity of IPEC-J2 cells. Values are stated as the mean ± SD. ^^^ *p* < 0.001 ZEN (72 h) versus control. ### *p* < 0.001 ZEN (48 h) versus control. * *p* < 0.05, *** *p* < 0.001 ZEN (24 h) versus control.

To illustrate the effects of ZEN on the activity of intestinal epithelial cells, the viability of IPEC-J2 cells after the action of different concentrations of ZEN (0, 60, 80, 100, 120, 140, 160, and 180 µM) and after incubation for different times (24 h, 48 h, and 72 h) was measured using the cell counting kit-8 (CCK-8) method. The results are shown in Figure 2; compared with the control group, three concentrations of ZEN (140, 160, and 180 µM) significantly reduced cell viability at 24 h (*p* < 0.05). Similarly, cell viability was also significantly reduced at 48 h (*p* < 0.001). Consecutively, cell viability was 66.23% at the concentration of 160 µM, and the 50% inhibitory concentration (LC50) was 175.3 ± 0.1 µM.

#### 2.2.2. Alkaline Phosphatase Activity and The Permeation of FITC-Dextran

**Figure 3:**
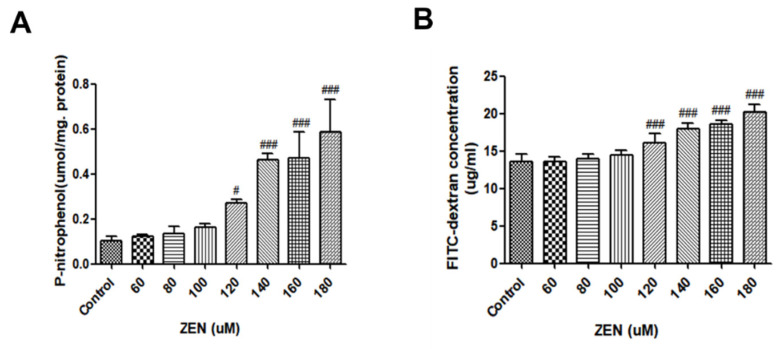
Effects of ZEN on permeability barrier function and intestinal integrity. (**A**) Effects of ZEN on AP activity in IPEC-J2 cells. (**B**) Effect of ZEN on FITC–dextran permeability in IPEC-J2 cells. Cells were treated with various concentrations of ZEN for 48 h. Data are expressed as means ± SD of three independent experiments. # *p* < 0.05, ### *p* < 0.001 ZEN (48 h) versus control.

Alkaline phosphatase (AP) is a ubiquitous enzyme distributed among many tissues and cell types, whose activity could reflect the cellular membrane integrity [26]. The IPEC-J2 cells were treated separately with different concentrations of ZEN for 48 h. As shown in Figure 3A, with increasing concentrations of ZEN, the higher the concentration of ZEN compared to the control, the higher the AP activity, and there was a relationship of dose between the concentration of ZEN and AP activity. Four concentrations of ZEN (120, 140, 160, and 180 µM) significantly increased the AP activity (*p* < 0.05). Therefore, our data demonstrated that cell membrane integrity was particularly sensitive to high concentrations of ZEN.

To further confirm the damage of ZEN to the monolayer epithelial cell barrier, the amount of penetration of FITC–dextran (4 kDa) across the IPEC-J2 cell monolayer was examined. Similarly, we treated IPEC-J2 cells separately with different concentrations of ZEN for 48 h. As seen in Figure 3B, four concentrations of ZEN (120, 140, 160, and 180 µM) significantly increased the concentration of FITC–dextran compared to the control (*p* < 0.001), excepting three concentrations (60, 80, and 100 µM). In following studies, based on this data, it was decided to treat the IPEC-J2 cells with 160~µM ZEN for 48 h.

### 2.3. Analysis of Transepithelial Electrical Resistance (TEER)

**Figure 4:**
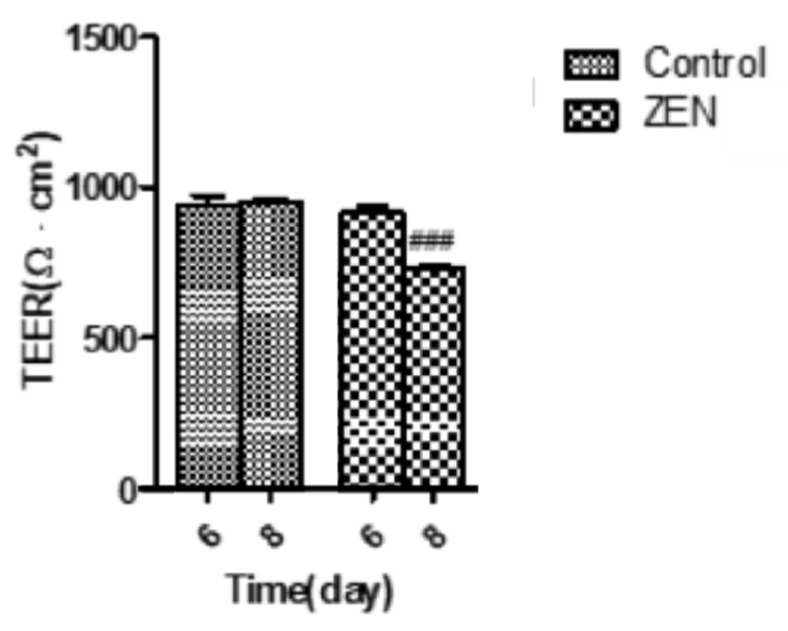
Effects of ZEN on TEER in IPEC-J2 cells. Values are expressed as means ± SD of three independent experiments. ### *p* < 0.001 ZEN (48 h) versus control.

From Figure 1, we get that the monolayer cell model was successfully constructed when the cells were grown up to day 6. Therefore, the IPEC-J2 cells were treated with ZEN (160 µM) on the 6th day after confluence. As seen in Figure 4, on day 6 there was no significant difference between the ZEN group and the control group. The TEER was measured again after 48 h of culture (8th day), and we found that the TEER of the ZEN treatment group (727.53 ± 11.85 Ω·cm^2^) was significantly decreased compared to the control group (953.23 261 ± 9.32 Ω·cm^2^) (*p* < 0.001). The results suggest that the decrease in TEER values of IPEC-J2 cells was caused by ZEN (Figure 4).

### 2.4. Effect of ZEN on IPEC-J2 Intracellular ATP

**Figure 5:**
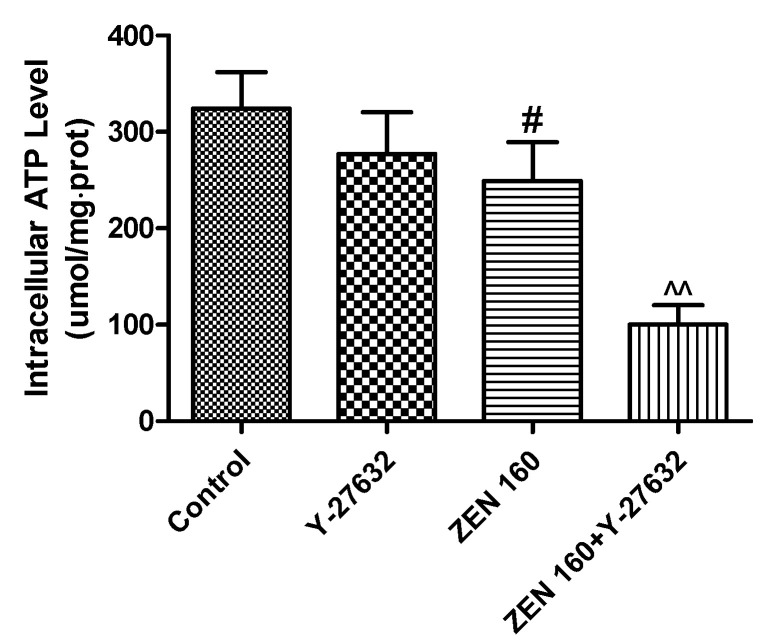
Effect of ZEN and Y-27632 on the intracellular ATP level in the IPEC-J2 cells. Values are expressed as means ± SD of three independent experiments. # *p* < 0.05 ZEN versus control; ^^ *p* < 0.01 ZEN versus ZEN + Y-27632.

To determine whether intracellular ATP acts on cytoskeletal remodeling, we next measured ATP levels in cells after 48 h of co-treatment with ZEN and Y-27632 (an ATP-competitive inhibitor of ROCK). As depicted in Figure 5, the intracellular ATP content of only Y-27632-treated cells was not significantly different from the control. However, after infection with ZEN, intracellular ATP content decreased significantly (*p* < 0.05). Additionally, the cellular ATP content of the Y-27632 and ZEN co-treated group was significantly lower than that of the group treated with ZEN alone (*p* < 0.01).

### 2.5. Effects of ZEN on IPEC-J2 Cytoskeletal Microfilament (F-Actin)

**Figure 6:**
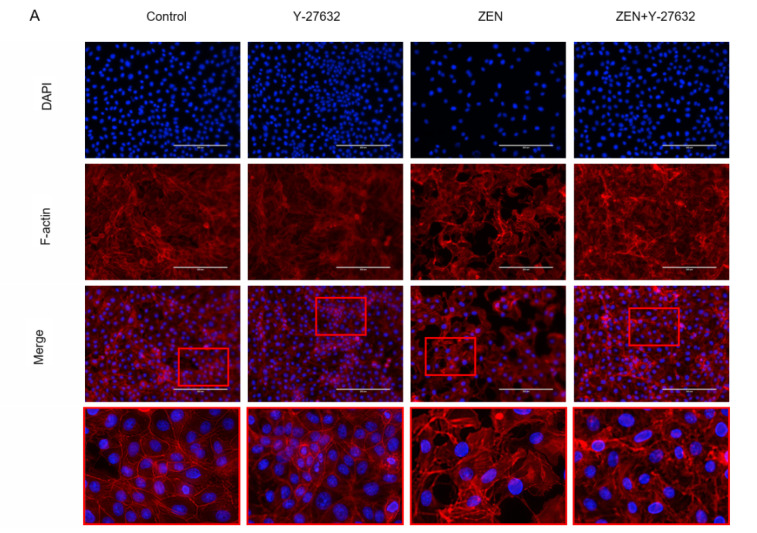

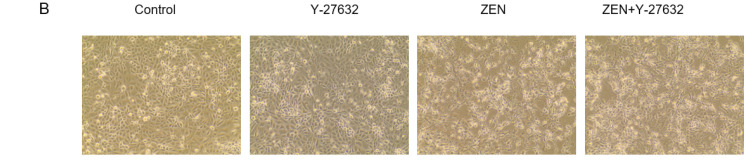
(**A**) Rhodamine-phalloidin staining of the IPEC-J2 cells monolayer. The phalloidin bound specifically to the cell’s fibronectin (F-actin) to exhibit orange-red fluorescence; the nuclei showed blue fluorescence after counterstaining with DAPI. Scale bar: 200 µm. (**B**) The morphology of the cells was observed under an inverted microscope. Magnification: 40 times.

The phalloidin bound specifically to the cell’s fibronectin (F-actin) to exhibit orange-red fluorescence. As seen in Figure 6A, the F-actin of the control group was dense, had a regular structure, and had a large number of pseudopods in the cells. Compared with the control group, Y-27632 pretreatment clearly modulated cytoskeletal rearrangements and showed a slight decrease in peripheral F-actin bundles in the IPEC-J2 cells. With the addition of ZEN, F-actin fluorescence was reduced and disordered, the central zone of the cell became less thick, the cells showed atrophy, and the number of cells was greatly reduced. In the ZEN 160 + Y-27632 group, the pretreatment of Y-27632 restored the disordered rearrangement of F-actin due to ZEN, the fluorescence was enhanced, and the number of cells was increased. As shown in Figure 6B, after ZEN treatment, the cell morphology changed, the intercellular space increased, most of them aggregated, and disintegration died. After Y-27632 pretreatment, the cell morphology was slightly improved, the intercellular gap became smaller, and the aggregation phenomenon was reduced.

### 2.6. Effects of ZEN on the Distribution and Expression of TJ Proteins

#### 2.6.1. The Expression mRNA of ROCK1 and Tight Junction Proteins

**Figure 7:**
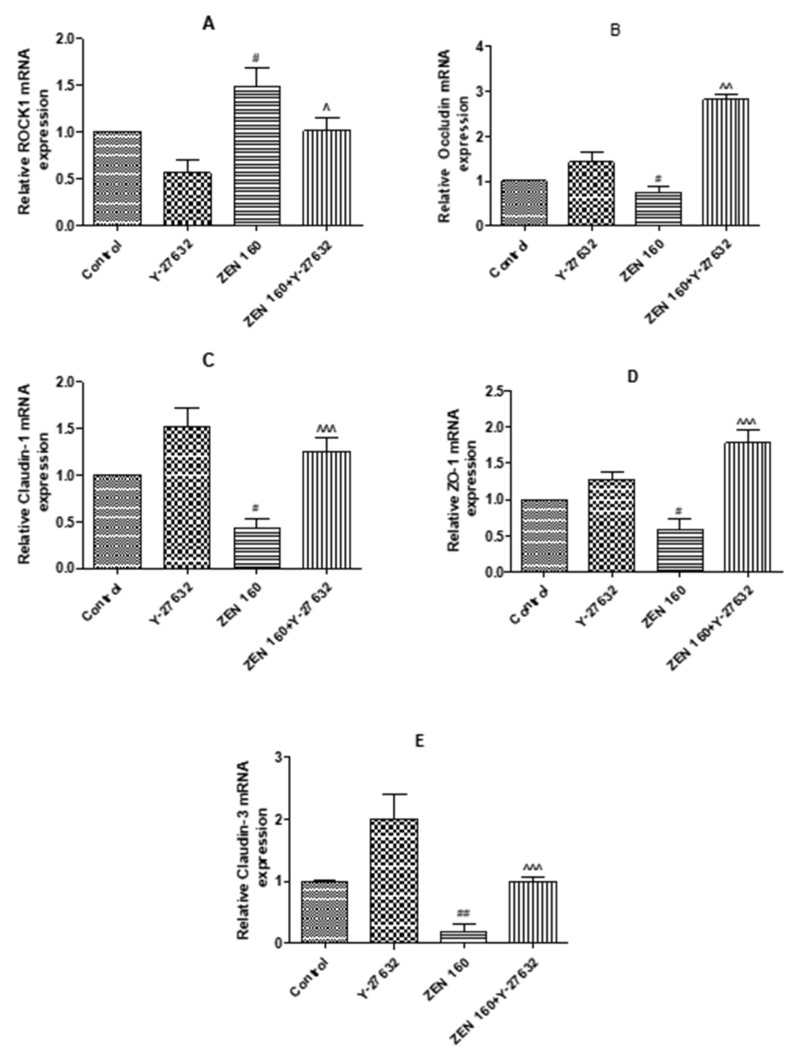
Effects of the ZEN exposure and Y-27632 pretreatment on mRNA expression levels of ROCK1 and tight junction-associated genes in the IPEC-J2 cells. (**A**–**E**) Data are means ± SD of three independent experiments. # *p* < 0.05, ## *p* < 0.01 ZEN versus control; ^ *p* < 0.05, ^^ *p* < 0.01, ^^^ *p* < 0.001 ZEN versus ZEN + Y-27632.

The functional integrity of the cytosolic barrier can be demonstrated by the expression of epithelial tight junction proteins. To explore the impacts of ZEN treatment on tight junction proteins, we examined the mRNA expression of four tight junction proteins (claudin-3, claudin-1, occludin, and ZO-1). As seen in Figure 7, the pretreatment of Y-27632 significantly increased the mRNA expression of four genes compared to the control (*p* < 0.05). The expression of cellular tight junction proteins at the transcriptional level was significantly lower in ZEN treatment than in the control group (*p* < 0.05). In the ZEN 160 + Y-27632 group, the pretreatment of Y-27632 significantly increased four different junction proteins (*p* < 0.05). Additionally, the transcript level of ROCK1 in the Y-27632-treated group was significantly lower than that in the control group (*p* < 0.05); ROCK1 was significantly increased after cells were exposed to ZEN (*p* < 0.05). Pretreatment with Y-27632 significantly improved transcript levels of ZEN-damaged tight junction proteins (*p* < 0.05).

#### 2.6.2. Immunofluorescence

**Figure 8:**
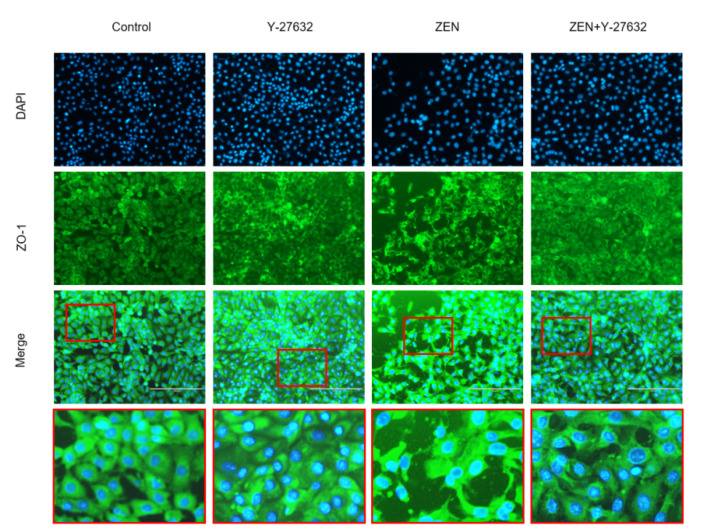
Morphology and expression of tight junction protein ZO-1 in IPEC-J2 cells. Cells were stained with antibodies for ZO-1 and detected by immuno-fluorescence after treatment. In the images that were collected, the nuclei showed blue fluorescence after counterstaining with DAPI, and ZO-1 exhibited green fluorescence around the nuclei. Scale bar: 200 µm.

We examined the effects of ZEN on ZO-1 protein expression and distribution using an immunofluorescent labelling assay (Figure 8), since the functional and structural integrity of intestinal epithelial cells can be reflected by the TJ protein (ZO-1). As seen in Figure 8, ZO-1 was located at the cell-cell contact zone in the control group. However, after ZEN exposure, the cell fluorescence brightness and expression distribution of ZO-1 was obviously decreased, intercellular junctions were unclear, and there was sporadic distribution of fluorescence rupture. In the ZEN 160 + Y-27632 group, the pretreatment of Y-27632 restored the fluorescence intensity of the ZO-1 protein, and the cell boundaries were slightly clearer.

### 2.7. Western Blotting

**Figure 9:**
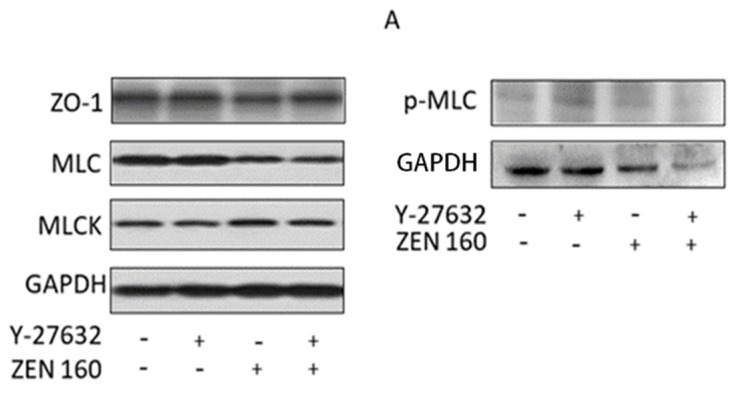

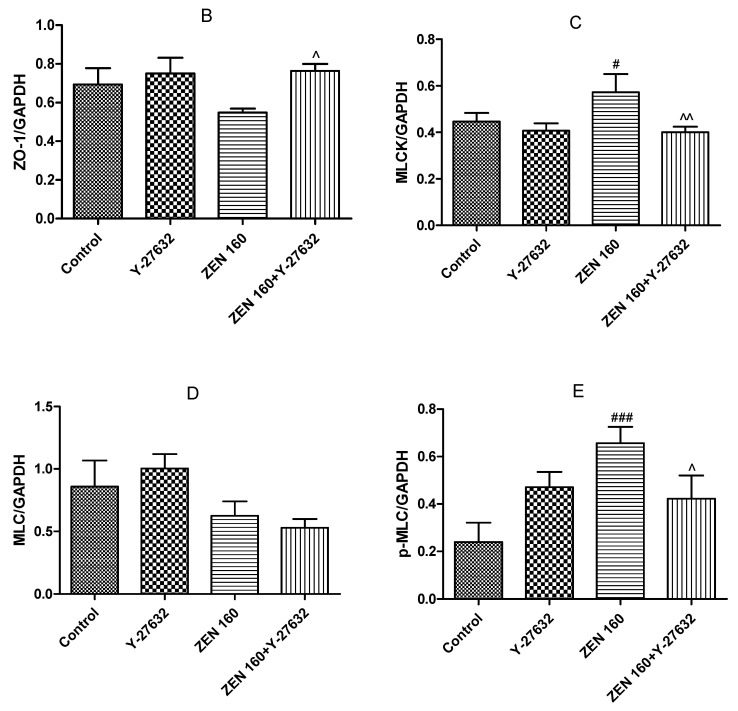
Effects of ZEN and Y27632 on protein expression levels of tight junction-associated protein and signaling pathway proteins in the IPEC-J2 cells. (**A**) Quantitative analysis of the expressions of the ZO1, MLCK, MLC, and p-MLC using Western blotting. (**B**–**E**) Values are means ± SD of three independent experiments. # *p* < 0.05, ### *p* < 0.001 ZEN versus control; ^ *p* < 0.05, ^^ *p* < 0.01 ZEN versus ZEN + Y-27632.

The related proteins of the RhoA/ROCK signaling pathway were detected by Western blotting. As seen in Figure 9A–E, in comparison with the control group, ZEN treatment had a tendency to reduce ZO-1 expression and to cause an increase in the protein expression of MLCK and p-MLC (*p* < 0.05). In contrast, the treatment group with Y-27632 resulted in increased expression levels of ZO-1, which was reduced by ZEN, and reduced the increased expression levels of MLCK and p-MLC caused by ZEN (*p* < 0.05).

## 3. Discussion

The intestinal mucosal barrier is an important intestinal mechanical barrier based on the intact intestinal mucosal epithelium and the tight junctions between intestinal epithelial cells. Under normal conditions, it effectively prevents harmful substances such as bacteria and endotoxins from passing through the intestinal mucosa into the bloodstream [27]. The measurement of TEER can reflect the state of tight junctions and allow the assessment of the role of boundary function in vitro [28,29,30]. We therefore allowed IPEC-J2 cells to grow on a permeable filter membrane scaffold, which was identified as a fused monolayer by trans-epithelial electrical resistance (TEER) measurements [31]. The measured values of TEER in the cells over a period of 18 days were monitored and stabilized to form a confluent monolayer model followed by ZEN treatment. In previous studies, when TEER ≥ 1000 Ω·cm^2^, it was considered that the single-layer confluence model was successfully established [32,33]. However, 500–1000 Ω·cm^2^ were obtained in this study; this observation was different from some earlier reports. The difference may be due to the serum concentration of the cell culture medium, which was confirmed by the research of Verhoeckx et al. [34]. As shown in Figure 1, on day 6, the TEER values of the IPEC-J2 cells entered a stable phase and the cells were still undergoing the differentiation process; therefore, drug-treated differentiated IPEC-J2 cells on day 6 were used as a model for the subsequent studies. Follow-up experiments on this basis revealed a gradual decrease in cell activity with increasing ZEN concentration and staining time, in a dose–time effect relationship. These findings are comparable to previous results [35]. Moreover, as compared to the control group, ZEN significantly reduced the TEER values, suggesting an influence of ZEN on IPEC-J2 cell barrier integrity.

Some research has demonstrated that disruption of epithelial cell integrity can cause increased cell permeability and disruption of cell barrier integrity [36,37]. The functional integrity of the cell membrane can be demonstrated by the measured AP activity values [26]. In the current study, we found that AP activity increased significantly with increasing ZEN exposure (Figure 3). Also, the ability of 4 kDa dextran to pass through the cellular bypass channel increased significantly with increasing ZEN concentration (Figure 3). This suggests that ZEN further compromises the destruction of cell barrier integrity by disrupting the integrity of the cell membrane.

TJs are complex in structure, being a complex of proteins [38]. Furthermore, if their structure and function are disrupted, it can lead to dysfunction of the corresponding mucosal barrier in animals and pose a threat to the health of the gastrointestinal tract [39]. There was evidence that when 200 ng-mL^−1^ deoxynivalenol was applied to the IPEC-J2 cells for 6 h, the presence of their tight junction proteins (claudin-1, occludin, and ZO-1) was significantly reduced, resulting in impairment of the mechanical barrier function of the intestine [40]. Therefore, to further demonstrate that ZEN exposure disrupts cell barrier function, we examined the expression of key tight junctions (TJs) in IPEC-J2 cells. The results showed that the transcript levels of tight junction proteins (ZO-1, occludin, and claudin-1) were significantly lower in the ZEN-treated cells than in the control cells. At the protein level, there was a tendency for ZEN treatment to reduce ZO-1 expression. The barrier function of epithelial cells depends on complex cytoskeletal organization, and transmembrane proteins and binding proteins in cells are linked to the cytoskeleton (microtubules, microfilaments, and silk) to regulate intestinal epithelial barrier function [41,42]. ZO-1 can bind to cytoskeleton, claudin-1, and occludin, and regulate the assembly of cytoskeletal dynamics at cell junctions [43]. Any change in the structure, function, or location can lead to disruption of tight junction protein structure, subsequently increasing intestinal mucosal permeability [44]. Based on these findings, the cytoskeletal state of rearrangement was observed after ZEN exposure by immunofluorescence and phalloidin staining in this study. Our results showed that ZEN induced severe rearrangement of cell microfilaments and the appearance of a number of vesicles. ZO-1 and F-actin were redistributed and there was cytoskeletal remodeling after ZEN treatment (Figure 6 and Figure 8). These results were consistent with the observed disruption of intercellular junctions and rearrangement of the cytoskeleton during intestinal infection or inflammation [45,46]. The above results suggest that ZEN contributes to the development of mechanical damage to the intestinal barrier.

Rho-related protein kinases (ROCKS) are key downstream effectors of RhoA420 signaling and play an important role in the actomyosin cytoskeleton, remodeling the cytoskeleton and maintaining the dynamic structure of intercellular junctional integrity [47,48]. Intestinal barrier dysfunction induced by disruption of the apical tight junction complex is associated with increased ROCK activity [49,50]. Alevizopoulos et al. found that the suppression of ROCK could weaken myosin phosphorylation and F-actin rearrangement and contraction [51]. Therefore, in this study, the transcript levels of ROCK1 were measured after ZEN treatment and were found to be significantly higher than those of the control cells. As shown in Figure 6B and Figure 9C,E, ZEN also leads to increased phosphorylation of the regulatory factors MLCK and MLC downstream of RhoA, which increases cell contractility, disrupts the integrity of the cell barrier, and leads to massive cell death (Figure 6B and Figure 9C,E). Severe rearrangement of cellular microfilaments and the appearance of a large number of vesicles were observed following ZEN treatment. We confirmed that ZEN activated the RhoA/ROCK signaling pathway, while causing mechanical damage to the intestinal barrier (Figure 6A).

To further confirm the relationship between mechanical damage to the intestinal barrier caused by ZEN and the RhoA/ROCK signaling pathway, we performed experiments with Y-27632 (an Rho/ROCK-specific inhibitor). Y-27632 inhibited MLCK phosphorylation in T24 and 5637 cells [52]. Similarly, we also found that Y-27632 (Figure 9C) significantly inhibited the ZEN-induced elevation of MLCK and activation of p-MLC. After pretreatment with Y-27632, cell morphology became longer, microfilaments increased, and the improper realignment was corrected (Figure 6A,B).

## 4. Materials and Methods

### 4.1. Chemicals and Reagents

Zearalenone (ZEN) was bought from Sigma-Aldrich (St. Louis, MO, USA). Fetal bovine serum (FBS) was obtained from Gibco-Life Technology (Grand Island, NY, USA). DMEM-F:12 cell culture medium was purchased from Thermo Fisher (Hyclone, China). CCK-8 was supplied by Dojindo (Kumamoto, Japan). Alkaline phosphatase (AP) activity assay kit, enhanced ATP assay kit, BCA assay kit, and ECL detection kit were supplied by Beyotime Biotechnology (Nantong, China). The purified ZEN powder was dissolved in ethanol (pre-experimental screening, 0.13%) to a stock solution of 100 mM and stored at −20 °C.

### 4.2. Cell Culture Conditions

IPEC-J2 cells were donated by the China Agricultural University. Cells were grown in DMEM-F:12 supplemented with 10% FBS 1% penicillin and streptomycin and cultured at 37 °C in a humidified 5% CO_2_ incubator. The medium was changed after 24 h. When the cells were grown to 80–90% in space, they were washed twice with PBS and 1 × trypsin/EDTA was added for digestion and passage.

### 4.3. Transepithelial Electrical Resistance (TEER)

TEER was utilized as the indicator of epithelial barrier competence and integrity [53]. TEER was recorded by an epithelial voltohm meter (EVOM; World Precision Instruments, Sarasota, FL, USA) over a period of 18 days. The IPEC-J2 cells were plated on a collagen-coated Transwell^®^ polyester membrane with a surface area of 1.12 cm^2^ (Corning Inc., Corning, NY, USA). The values of TEER were expressed as Ω·cm^2^.

### 4.4. Cell Activity Assay

IPEC-J2 cells (0.8–1.0 × 10^5^ cells/mL) were grown in 96-well culture plates; each group had five multiple holes. The IPEC-J2 cells were treated with different concentrations of ZEN (0, 60, 80, 100, 120, 140, 160, 180, 114 M) for 24, 48, and 72 h, respectively. The medium was aspirated and 20 μL of CCK-8 solution was added to each well and incubated for 2 h at 37 °C. Cell viability was measured by absorbance at 450 nm emission wavelength with a microplate reader (Tecen Austria GmbH Untersbergatr, Salzburg, Austria).

### 4.5. Alkaline Phosphatase Activity

The functional integrity of the cell membrane can be demonstrated by the measured AP activity values. [26]. IPEC-J2 cells (0.8–1.0 × 10^5^ cells/mL) were grown in 96-well culture plates and treated with different concentrations of ZEN for 48 h, respectively. The AP activity was determined by an AP activity assay kit according to the manufacturer’s instructions. The absorbance of AP activity was measured using a microplate reader at a wavelength of 405 nm.

### 4.6. Measurements of Paracellular Permeability

FITC-labeled dextran (4 kDa, Sigma, MO, USA) was used to measure the paracellular permeability. IPEC-J2 cells were grown in inserts and treated with ZEN as described in the previous section. The ZEN-treated IPEC-J2 cells were washed with PBS. Then, 1 mL 37 °C preheated HBSS (Gibco-Life Technology, Grand Island, NY, USA) was added to the bottom chambers; 100 µL FITC-dextran (final concentration 0.2 mg/mL) was added to the upper chambers. After 3 h incubation with the FITC–dextran at 37 °C, 100 µL samples were collected from the bottom chamber of the Transwell system. Absorbance was detected by a fluorescence spectrophotometer (excitation wavelength 480 nm, emission wavelength 520 nm). A standard curve was plotted, and the concentration of FITC-labeled dextran was calculated based on the absorbance values of the samples measured by the fluorescence spectrophotometer.

### 4.7. Design of RhoA/ROCK Signaling Pathway Validation Experiment

For all experiments, the cells were incubated until reaching confluence. The treatment groups were treated with drugs: control group; Y-27632 group (treated with 20 µM Y-27632 for 0.5 h before adding the same medium as the control group); ZEN group (160 µM ZEN for 48 h); ZEN + Y-27632 group (pre-treated with 20 µM Y-27632 for 0.5 h before adding the medium containing 160 µM ZEN). This experiment was designed for subsequent studies.

### 4.8. Quantitative Real-Time PCR

The cells were seeded in 6-well plates (2.0–2.5 × 10^6^ cells/mL) and treated as described in the previous section. Total RNA was extracted from cells by using TRIzol reagent (Invitrogen, Shanghai, China). The RNA concentration and purity were tested using a Nano Photometer P-Class (IMPLEN, Germany). RNA meeting the criteria was amplified into cDNA according to the SYBR Premix Ex Taq RT-PCR kit (Takara, Dalian, China) instructions. SYBR Green I RT-PCR kit (Takara, Dalian, China) was used to measure the mRNA expression of tight junction associated proteins (claudin-1, claudin-3, occludin, and ZO-1) and the signaling pathway-associated protein (ROCK1). The internal reference was GAPDH. Gene-specific primers (Table 1) were synthesized by Sangon (Shanghai, China).

RT-PCR was performed by an ABI PRISM 7500 SDS thermal cycler (Applied Biosystems, CA, USA). The relative expression of mRNAs was measured by the 2^−ΔΔCt^ method.

### 4.9. Immunofluorescence Staining

IPEC-J2 cells were inoculated in confocal dishes (2.0–2.5 × 10^6^ cells/mL) and treated as described in the previous section. After treatment, cells were washed 3 times with PBS. Afterward, cells were fixed with 4% paraformaldehyde for 30 min and 0.2% Tritonx-100 for 10 min. Subsequently, 2% BSA-PBS was added dropwise and blocked for 60 min at 37 °C. The cells were incubated with rabbit anti-ZO-1 (sc-33725, 1:500; Santa Cruz Biotechnology) primary antibody overnight at 4 °C, followed by incubation with Alexa Fluor-coupled anti-rabbit secondary antibody 169 (1:200; Beyotime Institute of Biotechnology, Shanghai, China) for 120 min at 37 °C. The cells were incubated with DAPI (Beyotime Institute of Biotechnology, China) for 30 s. Final photographs were taken under a fluorescence microscope (Life Technologies Corp., Bothell, WA, USA).

### 4.10. Intracellular ATP Determination

The cells were seeded in 6-well plates (2.0–2.5 × 10^6^ cells/mL). After being treated as described in the previous section, subsequent experiments were performed on ice packs throughout. The cells were washed 3 times with pre-cooled PBS. Pre-chilled cell lysis buffer was added and after they were fully lysed, centrifugation was performed. Intracellular ATP levels were measured in the supernatant using an enhanced ATP assay kit according to the manufacturer’s instructions.

### 4.11. Filamentous Actin (F-actin) Staining Filaments of Actin of F-Actin Analysis

Rhodamine-labeled phalloidin staining of monolayer cells was carried out to permit the visualization of the change of F-actin [54]. The cells’ treatment was consistent with the upper part treatment. After treatment, cells were washed with PBS and added to 4% paraformaldehyde fixative at room temperature for 10 min. The cells were treated with 0.5% Tritonx-100 for 5 min transparently. Then the cells were stained with 200 µL rhodamine-labeled phalloidin (TRITC) at room temperature for 30 min to label F-actin. Finally, DAPI (200 µL) was added to counterstain the nuclei for 30 s and photographed with a fluorescence microscope (Life Technologies Corp., Bothell, WA, USA) to compare the structure of the F-actin rim.

### 4.12. Western Blot Analysis

Cell sample proteins were collected according to the same lysis method described above. For the specific test operation steps, refer to Dong et al. [55]. After separation on an electrophoresis instrument (Bio-Rad, Hercules, CA, USA), the protein was transferred to a suitably sized PVDF membrane. The membrane was blocked with 5% BSA in TBS containing 0.05% Tween-20 at room temperature for 2 h and probed with the indicated primary antibodies at 4 °C overnight. After washing with TBST, goat anti-rabbit/mouse secondary antibody (Beyotime Institute of Biotechnology, Shanghai, China) was added at room temperature and incubated for 2 h. Proteins were detected by primary antibodies: ZO-1 (sc-33725, 1:1000; Santa Cruz Biotechnology), MLCK (1:2000; Beyotime Institute of Biotechnology, Shanghai, China), p-MLC and MLC (1:1000; Cell Signaling Technology, Boston, MA, USA). ECL chemiluminescent solution was added to the washed membranes and protein bands were detected on a gel recording system. The expression of each protein was normalized to that of GAPDH.

### 4.13. Statistics

Data plotting was performed using GraphPad Prime 5.0 software. Statistical figures were analyzed using SPSS 17.0 software (SPSS Inc., Chicago, IL, USA, 2008), and the data of three independent experiments were expressed as means ± SD. All the experimental data were analyzed for variance uniformity, and then analyzed by a one-way ANOVA (LSD). *p* < 0.05 was accepted as statistically significant.

## 5. Conclusions

Collectively, the present findings have provided important evidence that activation of the RhoA/ROCK signaling pathway is involved in ZEN-induced intestinal barrier dysfunction. ZEN exposure activated the RhoA/ROCK signaling pathway, increased p-MLC and remodeled the cytoskeleton (F-actin), reduced tight junction protein expression in turn, and improved the permeability of intestinal epithelial cells in IPEC-J2 cell monolayers, which finally induced intestinal barrier dysfunction. This study can provide a basis for further research on intestinal diseases associated with intestinal mucosal barrier injury and can provide a strong theoretical basis for the development and evaluation of new therapeutic drugs.

## Figures and Tables

**Table 1:** Primers used for qRT-PCR.

Genes	Orientation	Sequences (5′-3′)	Fragments Size (bp)	Accession Number
GAPDH	Forward	GATGGTGAAGGTCGGAGTGAAC	153	NM_001206359.1
	Reversed	TGGGTGGAATCATACTGGAACA		
Occludin	Forward	ACGAGCAGCAAAGGGATTCTTC	152	NM_001163647.2
	Reversed	TCACACCCAGGATAGCACTCATT		
Claudin-1	Forward	TGCCTCAGTGGAAGATTTACTCC	147	NM_001244539.1
	Reversed	TGGTGTTCAGATTCAGCAAGGA		
ZO-1	Forward	AGTTTGATAGTGGCGTTGACAC	106	XM_005659811.1
	Reversed	GCTGAAGGACTCACAGGAACA		
Claudin-3	Forward	GTCCATGGGCCTGGAGAT	130	NM_001160075.1
	Reversed	GATCTGCGCTGTGATAATGC		
ROCK1	Forward	TTGTGCCTTCCTTACTGACAGG	125	XM_005653400.3
	Reversed	CTGGTGCCACAGTGTCTCG		

## Data Availability

Not applicable.

## Abbreviations

ZEN, zearalenone; IC50, 50% inhibitory concentration; AP, alkaline phosphatase; TEER, transepithelial electrical resistance; MLC, myosin light chain; MLCK, myosin light chain kinase; ROS, reactive oxygen species; IECs, intestinal epithelial cells; TJs, tight junctions; HUVECs, human umbilical vein endothelial cells; ZOs, zonula occludens; ROCK, Rho-associated protein kinase; AJC, apical tight junction complex; ATP, adenosine triphosphate; MPTP, mitochondrial permeability transition pore; PM, plasma membrane; CCK-8, cell counting kit-8.
